# ApCPEB4, a non-prion domain containing homolog of ApCPEB, is involved in the initiation of long-term facilitation

**DOI:** 10.1186/s13041-016-0271-x

**Published:** 2016-10-22

**Authors:** Seung-Hee Lee, Jaehoon Shim, Ye-Hwang Cheong, Sun-Lim Choi, Yong-Woo Jun, Sue-Hyun Lee, Yeon-Su Chae, Jin-Hee Han, Yong-Seok Lee, Jin-A Lee, Chae-Seok Lim, Kausik Si, Stefan Kassabov, Igor Antonov, Eric R. Kandel, Bong-Kiun Kaang, Deok-Jin Jang

**Affiliations:** 1 Department of Biological Sciences, College of Natural Sciences, Seoul National University, 1 Gwanangno, Gwanak-gu Seoul, 08826 South Korea; 2 Department of Biological Sciences, KAIST, Daejeon, 34141 South Korea; 3 Department of Ecological Science, College of Ecology and Environment, Kyungpook National University, 2559, Gyeongsang-daero, Sangjusi, Gyeongsangbuk-do 37224 South Korea; 4 Department of Bio and Brain Engineering, KAIST, Daejeon, 34141 South Korea; 5 Department of Physiology, College of Medicine, Seoul National University, Seoul, 03080 South Korea; 6 Department of Biotechnology and Biological Science, College of Life Science and Nano Technology, Hannam University, Daejeon, 34054 South Korea; 7 Stowers Institute for Medical Research, Kansas City, MO 64110 USA; 8 Howard Hughes Medical Institute, 1051 Riverside Drive, New York, NY 10032 USA; 9 Department of Neuroscience, New York State Psychiatric Institute, Kavli Institute for Brain Sciences, Columbia University College of Physicians and Surgeons, New York, NY 10032 USA

**Keywords:** *Aplysia*, Long-term facilitation, CPEB, CPEB4

## Abstract

Two pharmacologically distinct types of local protein synthesis are required for synapse- specific long-term synaptic facilitation (LTF) in *Aplysia*: one for initiation and the other for maintenance. ApCPEB, a rapamycin sensitive prion-like molecule regulates a form of local protein synthesis that is specifically required for the maintenance of the LTF. However, the molecular component of the local protein synthesis that is required for the initiation of LTF and that is sensitive to emetine is not known. Here, we identify a homolog of ApCPEB responsible for the initiation of LTF. ApCPEB4 which we have named after its mammalian CPEB4-like homolog lacks a prion-like domain, is responsive to 5-hydroxytryptamine, and is translated (but not transcribed) in an emetine-sensitive, rapamycin-insensitive, and PKA-dependent manner. The ApCPEB4 binds to different target RNAs than does ApCPEB. Knock-down of ApCPEB4 blocked the induction of LTF, whereas overexpression of ApCPEB4 reduces the threshold of the formation of LTF. Thus, our findings suggest that the two different forms of CPEBs play distinct roles in LTF; ApCPEB is required for maintenance of LTF, whereas the ApCPEB4, which lacks a prion-like domain, is required for the initiation of LTF.

**Electronic supplementary material:**

The online version of this article (doi:10.1186/s13041-016-0271-x) contains supplementary material, which is available to authorized users.

## Introduction

Unlike short-term memory, long-term memory requires new protein synthesis for its formation [1–7]. Protein synthesis occurs in two spatially distinct regions of the neuron: 1) in the cell body where activity-dependent transcription and subsequent translation occurs and 2) in the presynaptic terminals and in the postsynaptic dendritic spines where mRNAs are localized and translated following synaptic activation [8–10]. The second form of translation is responsible for local protein synthesis, which is important for both the initiation and the maintenance of long-term memory.

The cytoplasmic polyadenylation element binding protein (CPEB) has been identified as one key regulator of the local protein synthesis in *Aplysia* [6]. The binding of CPEB to mRNAs regulates the translation of target mRNAs by regulating their polyadenylation [11–14]. ApCPEB binds to the 3′ untranslated region (3′ UTR) of mRNAs that contains conserved cytoplasmic polyadenylation element (CPE) binding site (UUUUUAU) [15]. ApCPEB is locally activated in response to a single pulse of 5-hydroxytryptamine (5-HT) and is inhibited by rapamycin. Interestingly, ApCPEB has a prion-like domain that is important for the ability of ApCPEB to form aggregates that are self-sustaining and can maintain the increased level of ApCPEB proteins in the terminals that is critical for maintaining long-term facilitation (LTF) in *Aplysia* sensory-motor neuron synapse [15–17]. When the translation of the ApCPEB mRNA is blocked locally, the initiation of LTF at 24 h is intact, whereas the maintenance of LTF at 72 h is selectively and specifically impaired. One of the major mRNA targets of ApCPEB is the actin mRNA, which contains the CPE site on its 3′ untranslated region (3′UTR) and is locally translated during LTF [15]. ApCPEB has two isoforms, one contains poly-Q prion domain and the other lacking the prion-like domain [15, 18]. The maintenance of LTF requires the form of ApCPEB, which contains the prion domain.

In this study, we identified a new CPEB protein, ApCPEB4, in *Aplysia kurodai*. This protein is homologous to the mammalian CPEB4. The level of expression of ApCPEB4 was increased by 5-HT in a translation-dependent manner. Unlike ApCPEB, ApCPEB4 bound to specific RNA in a CPE-independent manner and is required for the initiation but not for the maintenance of LTF. Overexpression of ApCPEB4 reduced the threshold of the LTF induction. In addition, PKA-mediated phosphorylation of ApCPEB4 was critical for the induction of LTF. Collectively, these data suggest that ApCPEB4 plays a key role in regulating the initiation of LTF, while ApCPEB is essential for the maintenance of LTF.

## Methods

### Cloning of ApCPEB4 from *Aplysia kurodai*

**Fig. 1:**
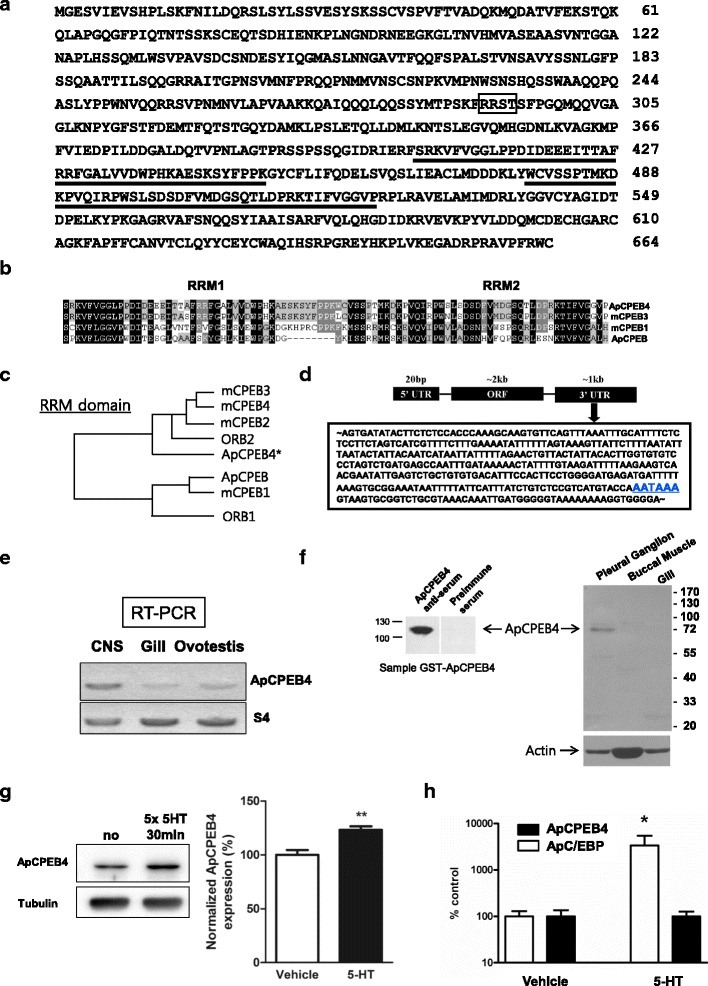
Cloning of ApCPEB4 and its expression in the CNS. **a** Amino acid sequence of a cloned full-length ApCPEB4. Sequence analysis showed that ApCPEB4 had two conserved RRMs (*underlined*), one conserved PKA phosphorylation sites (*box*). **b** Alignment of RRM domain of *Aplysia* CPEB4 (ApCPEB4), mouse CPEB3 (mCPEB3), mouse CPEB1 (mCPEB1) and *Aplysia* CPEB (ApCPEB). **c** The phylogenetic relationship between CPEBs in different species was determined by ClustalW. **d** mRNA structure of the ApCPEB4. ApCPEB4 contains ~20 bp 5′UTR (untranslated region), ~2 kb open reading frame (ORF), and ~1 kb 3′UTR. Arrowed inset indicates the detailed nucleotide sequence of the 3′UTR. Blue underline indicates hexanucleotide sequence (AATAAA). **e** Expression pattern of ApCPEB4 mRNA. RT-PCR of total RNA (1 μg) isolated from *Aplysia* CNS, ovotestis, or gill with gene-specific primers. *Aplysia* housekeeping gene S4 was used as a control for the amplification. **f** Western blotting of ApCPEB4 using purified GST-fused ApCPEB4 or total lysates from various tissues including pleural ganglion, buccal ganglion and ovotestis. **g** A representative Western blot (*left*) and quantification (*right*) of ApCPEB4 in *Aplysia* pleural ganglia extracts prepared from pleural-to-pedal ganglia exposed to 5 times of 5 min treatment of 5-HT. Total extracts were prepared at indicated times and 20 μg of proteins were blotted with anti-ApCPEB4 antibodies (*left, top panel*). The same extracts were also blotted with anti-tubulin antibodies as loading controls (*left, bottom panel*). 5-HT treatment significantly increased the level of ApCPEB4 in the extracts. **, *p* < 0.01, two-tailed unpaired *t* test. **h** One microgram of total RNA from pleural ganglia was used for RT-PCR with gene-specific primers. As a stimulation control, we used ApC/EBP, an immediate early gene. ApC/EBP was transcriptionally enhanced in response to 5-HT stimuli. *Aplysia* S4 was used as an amplification and loading control. *, *p* < 0.05 compared to that of control ApC/EBP, two-tailed unpaired *t* test

We obtained the ApCPEB4 fragment of *Aplysia kurodai* from EST database by searching through custom-made basic local alignment software. Using this fragment as a probe, we screened ~1.5 × 10^5^ clones of an *Aplysia kurodai* cDNA library and isolated several clones encoding parts of ApCPEB4. Based on the sequences of these clones, we obtained the full length of ApCPEB4. The length of coding region was 2064 bp and 664 amino acids, and it also contained two RNA Recognition Motifs (Fig. 1a). Using Expasy software (http://www.expasy.org/), potential PKA phosphorylation sites were searched.

3× CPE or CPE mutant sites were obtained by PCR with specific primer sets: 3× CPE1, CPE1-D3-S (5′-CGCCCAAGCTTGCAGCTTTTTATGACACAC AGT TTTTATGATGCCACG-3′)/CPE1-EI-A (5′-GCATGAATTCGATGGATAAAAACGTGGCA CATAAAAACTGTGTGTC-3′); 3× CPE2, CPE2-D3-S (5′-CGCCCAAGCTTGCAGCTT TTA ATG ACA CAC AGT TTT AAT GAT GCC ACG-3′)/CPE2-EI-A (5′-GCA TGA ATT CGATGGATTAAAACGTGG CATCATTAAAACTGTGTGTC-3′); 3× CPE3, CPE3-D3-S (5′-CGCCCAAGCTTGCAGCTTTTATAAGGACACACAGTTTTATAAGGATGCCACG-3′)/CPE3-EI-A (5′-GCATGAATTCGATGGCTTATAAAACGTGGCATCCTTATAAAA CTGTGTGTC-3′); 3× CPEmt1, CPEmt1-D3-S (5′-CGCCCAAGCTTGCAGCTTTTTGTG ACACACAGTTTTTGTGATGCCACG-3′)/CPEmt1-EI-A (5′-GCATGAATTCGATGGACA AAAACGTGGCATCACAAAAACTGTGTGTC-3′); 3× CPEmt2, CPEmt2-D3-S (5′-CGC CCAAGCTTGCAGCTT TTTGGTGACACACAGTTTTTGGTGATGCCACG-3′)/CPEmt2-EI-A (5′-GCATGAATTCGATGGACCAAAAACGTGGCATCACCAAAAACTGTGTGTC-3′). The PCR products were separately sub-cloned into *Hind*III–*EcoR*I-digested pcDNA3.1(+) to create pcDNA3.1-3×CPEs.

### Kinase assays

A kinase assay was carried out at 30 °C for 30 min in a final volume of 25 μl of reaction buffer (50 mM Tris-Cl, 10 mM MgCl_2_, pH 7.5) containing 1 μg substrate, 200 μM ATP, 1 mCi [γ^32^P]ATP and 5 units of PKA catalytic subunit (NEB). Reactions were stopped by adding SDS-sample buffer and boiling at 100 °C for 5 min. Then, [^*32*^ *P*] phosphate incorporation was analyzed by SDS-PAGE and a phosphoimager. To confirm the specificity of phosphorylation by PKA, either 40 μM KT5720 (AG Science) or dimethyl sulfoxide (DMSO) (Sigma) was added to the reaction mixture.

To examine whether ApCPEB4 is an endogenous substrate of *Aplysia* PKA, the crude tissue extract from *Aplysia* pedal-pleural ganglia was prepared as previously described [19]. The reaction was carried out at 18 °C for 20 min containing GST-agarose bead binding 1 μg of GST-ApCPEB4, 10 μg of tissue extract and 1 mCi [γ^32^P]ATP in extraction buffer. To confirm the specificity of phosphorylation, the crude tissue extracts were incubated with inhibitors of specific kinases, 40 μM KT5720 (PKA inhibitor) [20], 20 μM PD98059 (MEK inhibitor) or 10 μM chelerythrin (PKC inhibitor), for 10 min. A GST-pull down assay was performed as previously described [21]. The [^*32*^ *P*] phosphate incorporation was analyzed by SDS-PAGE and a phosphoimager.

### Recombinant protein purification and antibody production

For the antibody production, the N-terminal 400 bp of ApCPEB4 was amplified by PCR and ligated into pRSETa (Invitrogen), a His-tag vector. The His-ApCPEB4-N protein expression was induced by 2 mM IPTG for 3 h at 37 °C and purified by a Ni-NTA purification system (Invitrogen). Polyclonal anti-ApCPEB4 antibodies were raised in mice using this purified protein. The peptide competition assay was performed by western blot using the ApCPEB4 antibodies incubated with either 25 μg of purified His-ApCPEB4-N or 25 μg of BSA as a control at 4 °C overnight.

### RT-PCR, western blot, and immunocytochemistry

To examine the expression of ApCPEB4, an RT-PCR was performed using the total RNAs from various *Aplysia* tissues or HEK293T cells using gene-specific primers. For loading control, PCR was performed against S4 for *Aplysia*. For the induction control, PCR was performed against *Aplysia* CCAAT-enhancer-binding proteins (ApC/EBP). A western blot was performed in the pleural ganglia, buccal muscle, and gill extracts. Anti-ApCPEB4, and anti-actin antibodies were used to detect each protein within the same loaded sample. To examine the induction level of ApCPEB4 in response to 5-HT, pleural-pedal ganglia were prepared in a sylgard plate and then applied with 5 pulses of 5-HT (20 μM for 5 min at 20 min interval). Pleural ganglia were prepared 30 min after final application of 5-HT. For the immunostaining of endogenous ApCPEB4, cultured neurons were washed with cold ASW twice and immediately fixed with 4 % paraformaldehyde in PBS after either the application of massed 5-HT (10 μM for 1 h) or 5 pulses 5-HT (10 μM for 5 min) at 20 min interval.. Fixed cells were washed with PBS and permeabilized with 0.2 % Triton X-100 in PBS for 10 min. After blocking with 3 % BSA (Amersham Biosciences, Piscataway, NJ) for 2 h at room temperature, primary antibodies were treated (1:500 of anti-ApCPEB4 serum) overnight at 4 °C. The cells were washed with PBS and treated with secondary antibody, Cy3-conjugated anti-mouse IgG (Amersham Biosciences, Piscataway, NJ) for 1 h at room temperature. Immunostained images were acquired by a confocal laser scanning microscope (LSM510, Carl Zeiss, Jena, Germany).

### mRNA-protein pull-down assay

mRNA-protein pull-down assay was performed as described previously [22] with small modification. Actin 3′UTR was obtained from *Aplysia* ganglion cDNA, and Luciferase-1904 (Luc-1904) was obtained by oligomer annealing and subcloned into pGL3UC vector (Promega) [23]. The biotin labeled RNA was prepared by in-vitro transcription with T7 RNA polymerase (Promega) using the nucleotide analog Bio-17-ATP and Bio-11-CTP (Enzo). Each biotinylated RNA was analyzed by agarose-gel electrophoresis and quantified by nano-drop. HEK293T cells overexpressing Flag-tagged target proteins were lysed using lysis & binding buffer containing 50 mM Tris–HCl (pH 7.6), 150 mM NaCl, 5 % glycerol, 0.1 % Triton X-100, 1 mM DTT, 0.2 mg/mL heparin, 0.2 mg/mL yeast tRNA, 0.25 % BSA, protease inhibitor cocktail (Roche), and 40 U/mL RNasin (Promega). 8 μg of biotinylated RNAs were mixed with pre-cleared 200 μg (0.2 mg/mL) of 293 T cell lysate and incubated on a rotator for 1 h at 4 °C. 30 μl of NeutraAvidin Agarose Resin (Thermo) was added to each tube, and the mixture was further incubated for 2 h. Beads were washed five times with washing buffer containing 50 mM Tris–HCl (pH 7.6), 150 mM NaCl, 5 % glycerol, 0.1 % Triton X-100, 1 mM DTT and 40 U/mL RNasin. Western blots were performed with mFlag-M2 antibody (1:2000, Sigma).

### Cell cultures and microinjection

Primary culture of *Aplysia* sensory neurons and coculture of sensory-to-motor neurons were made as described previously [24–26]. Briefly, Abdominal and central ganglia were dissected from *Aplysia kurodai* (50-100 g) and incubated at 34 °C for 1.5 ~ 2.5 h in 1 % protease (type IX, Sigma) dissolved in isotonic L15/ASW (1:1) media (ASW: 460 mM NaCl, 10 mM KCl, 11 mM CaCl_2_, 55 mM MgCl_2_, and 10 mM HEPES, pH 7.6). After a thorough washing with ASW several times to remove residual protease, the ganglia were incubated at 18 °C for at least 3 h in L15/ASW to allow for recovery from heat shock. LFS motor neurons were dissected from the abdominal ganglia and cultured in a solution of 50 % *Aplysia* hemolymph in isotonic L15 media. The next day, pleural sensory neurons were isolated from the pleural ganglia and cocultured with LFS motor neurons and maintained at 18 °C in an incubator for 3 days to allow time for the formation and stabilization of synaptic connections. Microinjection of DNAs and double-strand RNAs into *Aplysia* neurons was done by air pressure as described elsewhere [27, 28].

### Electrophysiology

The LFS motor neuron was impaled with a glass microelectrode filled with 2 M K-acetate, 0.5 M KCl, 10 mM K-HEPES (10–15 MΩ), and the membrane potential was held at −80 mV. The excitatory postsynaptic potential (EPSP) in the motor neuron was evoked by stimulating the sensory neurons with a brief depolarizing stimulus using an extracellular electrode. The initial EPSP value was measured 24 h after microinjection. The cultures then received one pulse or five pulses of 10 μM 5-HT for 5 min at 15-min interval to induce LTF. The amount of synaptic facilitation was calculated as a percentage change in EPSP amplitude recorded after the 5-HT treatment compared with its initial value before treatment.

## Results

### Cloning of ApCPEB4-like protein, a homologue of mammalian CPEB4

As an initial step in investigating the role of other CPEBs in *Aplysia*, we obtained an expressed sequence tag (EST) clone homologous to the conserved RNA recognition motif (RRM) of mammalian CPEB2-4 family from the *Aplysia kurodai* EST database [29]. Using this EST clone as a probe, we carried out a library screening and cloned a full-length cDNA of a novel *Aplysia* CPEB (Fig. 1a). We named the clone ApCPEB4 as it is 99 % identical to CPEB4-like gene in the genomic database of *A. californica* (NCBI accession #, XP005089812). ApCPEB4 has a unique N-terminus and two conserved RRM on the C-terminus [15, 30] (Fig. 1a). Unlike the long form of ApCPEB, which was cloned previously [18], ApCPEB4 does not have a prion poly-Q domain. ApCPEB4 has a potential PKA phosphorylation site (RRST, consensus sequence (RRX(S/T)) outside the RRM domains (Fig. 1a). Even though the sequence was not identical, the overall phylogenetic analysis of the phosphorylation site and the RRM domain of ApCPEB4 revealed that ApCPEB4 is homologous to mammalian CPEB2-4 and Drosophila Orb2 (Fig. 1b and c). The amino acid sequences of the ApCPEB4 RRM domain are 83.0 % identical to mouse CPEB2, 82.0 % to mouse CPEB3, 80.7 % to mouse CPEB4, 77.4 % to Orb2, 34.4 % to mouse CPEB1, 32.7 to Orb1 and 31.0 % to ApCPEB, respectively. These analyses suggest that ApCPEB4 is homologous to the members of the mammalian CPEB2-4 family. Interestingly, the ApCPEB4 3′ untranslated region (UTR) (~1 kb) contains the nuclear polyadenylation hexanucleotide sequence (Fig. 1d).

We next examined the expression of ApCPEB4 in various *Aplysia* tissues by Reverse Transcription-Polymerase Chain reaction analysis (RT-PCR). ApCPEB4 was expressed in the extracts of central nervous system (CNS) and other tissues including gill and ovotestis (Fig. 1e). Western blot analysis detected significant bands with the size of ~100 kDa and ~70 kDa in both purified proteins and protein extracts from *Aplysia* pleural ganglia, respectively (Fig. 1f). Taken together, these data indicate that ApCPEB4 is another neuronal CPEB protein that belongs to CPEB family in *Aplysia*.

### ApCPEB4 is synthesized in response to 5-HT signaling

We next asked whether the expression of ApCPEB4 is regulated in response to 5-HT. We found that the level of ApCPEB4 protein in the ganglia extracts was significantly increased by either spaced (5 times pulses of 5 min each) (Fig. 1g) or massed (2 h) application of 5-HT onto the intact pleural-to-pedal ganglia, both of which are known to induce long-term facilitation (Additional file 1: Figure S1). The increase in protein level was not transcription-dependent, because ApCPEB4 RNA transcript was not increased by 5-HT treatment (Fig. 1h).

**Fig. 2:**
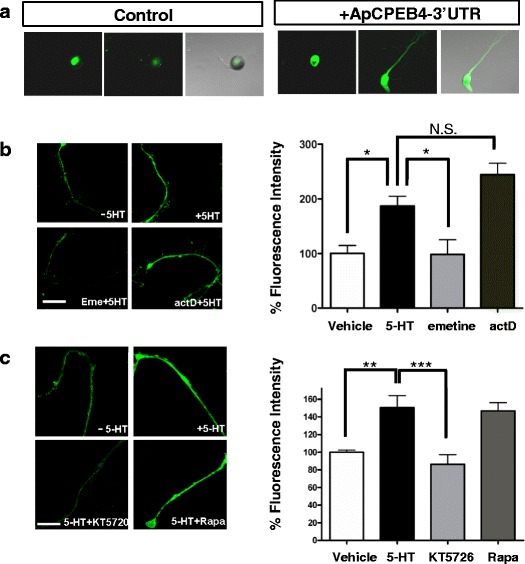
ApCPEB4 expression is increased by the activation of 5-HT signaling in the isolated neurites. **a** ApCPEB4 3′UTR enhances local translation of reporter cDNA. The reporter gene nGFP (nuclear GFP) expression, which normally occurs in the nucleus (control), was observed at the distal neurite in the presence of the 3′UTR of ApCPEB4 (+ApCPEB4-3′UTR). **b** Immunostaining for ApCPEB4 showed significant induction of ApCPEB4 following 5-HT application in the isolated neurites. The induction of ApCPEB4 was blocked by concurrent treatment of emetine, not by actinomycin D (actD). *, *p* < 0.05; n.s., not significant, one-way ANOVA; F = 12.73, Tukey’s post-hoc test. **c** Concurrent treatment of KT5720, a PKA inhibitor, significantly blocked the induction of ApCPEB4 following 5-HT treatment, while the rapamycin (rapa), a blocker for mTOR-dependent protein translation, has no effect on the ApCPEB4 induction. **, *p* < 0.05; ***, *p* < 0.001; one-way ANOVA; F = 9.23, Tukey’s post-hoc test

Transcription-independent increase of ApCPEB4 suggests that 5-HT signaling may regulate translation of ApCPEB4 mRNA or stability of ApCPEB4 protein. We first examined whether ApCPEB4 mRNA was present and localized at the distal neurite. When the 3′UTR of ApCPEB4 was added at the end of the cDNA sequence of a reporter gene - nGFP (nuclear GFP)- the GFP signal was observed at the distal neurite (Fig. 2a). This supports the idea that the 3′UTR of ApCPEB4 is sufficient for the localization and translation of the mRNA at the distal neurite. We next cut off the cell bodies of cultured sensory neurons, and stimulated the isolated neurites for 1 h with 10 μM 5-HT. We found that ApCPEB4 immunoreactivity was increased about 2 fold in the stimulated neurites compared with neurites treated with vehicle- (vehicle, 100.0 ± 14.4 %, *n* = 6 versus 5-HT, 186.8 ± 17.8 %, *n* = 6; * *p* < 0.05, one-way ANOVA; F = 12.73, Tukey’s post-hoc test.) (Fig. 2b). This increase is also observed in the neurites treated with pulsed 5-HT (5 min of 10 μM 5-HT, 5 times; vehicle, 100.0 ± 46.1 %, *n* = 43 versus 5×5-HT, 128.8 ± 5.9 %, *n* = 60; two-tailed unpaired *t* test, *p* < 0.01). The up-regulation of ApCPEB4 was blocked by emetine (100 μM), a non-selective protein synthesis inhibitor (vehicle, 100.0 ± 14.4 %, *n* = 6; 5-HT, 186.8 ± 17.8 %, *n* = 6; emetine, 98.24 ± 26.9 %, *n* = 5; *, *p* < 0.05; n.s., not significant; one-way ANOVA; F = 12.73, Tukey’s post-hoc test) (Fig. 2b). Conversely, the induction of ApCPEB4 was not affected by the transcriptional inhibitor, actinomycin D (50 μM) (actD, 244.3 ± 20.7 %, *n* = 7; *p* > 0.05, one-way ANOVA; F = 12.73, Tukey’s post-hoc test.) (Fig. 2b). These results together suggest that 5-HT signaling enhances translation, but not transcription of ApCPEB4 mRNA in the stimulated neurites.

Two distinct translational mechanisms are known to be recruited during 5-HT-mediated synaptic facilitation in *Aplysia*: rapamycin-sensitive and -insensitive ones [31]. Since ApCPEB4 was translated in the isolated neurites, we further tested whether this translational induction is sensitive to the rapamycin. When the rapamycin (20 nM) was added together with 5-HT on the isolated neurites, translational induction of ApCPEB4 was not blocked, indicating that translation of ApCPEB4 is rapamycin-insensitive (Fig. 2c). Rapamycin-insensitive, but emetine-sensitive local translation requires protein kinase A (PKA) activity for the initiation of synapse-specific LTF [31, 8]. Translation of ApCPEB4 was blocked by KT-5720 (PKA inhibitor, 5 μM) (Fig. 2c), raising the possibility that the translation of ApCPEB4 might be critical for the initiation of LTF (vehicle, 100.0 ± 2.5 %, *n* = 10 versus 5-HT, 150.4 ± 13.7 %, *n* = 14; ** *p* < 0.05; KT-5720, 86.4 ± 10.8 %, *n* = 13; rapamycin, 146.7 ± 9.5 %, *n* = 13; *, *p* < 0.05; ***, *p* < 0.001; one-way ANOVA; F = 9.23, Tukey’s post-hoc test).

### RNA binding specificity of ApCPEB4

**Fig. 3:**
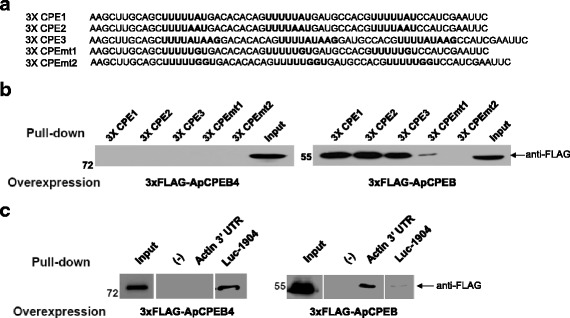
RNA binding specificity of ApCPEB4 and ApCPEB. **a** RNA sequences of CPE1, CPE2, CPE3, CPEmt1, and CPEmt2. **b** A full-length of ApCPEB4 did not bind to any CPEs and CPEmts (*left*), whereas a full-length of ApCPEB significantly bound to CPE1, CPE2 and CPE3 but not to CPEmt1 and CPEmt2 (*right*). **c** A full-length of ApCPEB4 only bound to 1904 sequence but not to 3′ UTR of both neuronal actin (*left*). On the other hand, a full-length of ApCPEB bound to 3′ UTR of neuronal actin, but not to 1904 sequence (*right*)

A growing body of evidence suggests that mammalian CPEB1 and CPEB2-4 family have different target RNAs. For example, CPEB1 has higher affinity to CPE site on the 3′UTR of target mRNAs, but CPEB3-4 are believed to recognize specific RNA secondary structure [23]. We tested whether *Aplysia* CPEB proteins, ApCPEB and ApCPEB4, also show difference in RNA binding properties. We first generated five different target RNA constructs containing three types of three-repeated (3×) CPE sites (CPE1 (UUUUUAU), CPE2 (UUUUAUU) and CPE3 (UUUUAUAAG) or two types of 3× CPE mutant sites (CPEmt1 (UUUUUGU) and CPEmt2 (UUUUUGGU)) (Fig. 3a). ApCPEB4 did not bind to any CPE or CPE mutant site, whereas ApCPEB bound to CPE sites but not to CPE mutant sites (Fig. 3b). These results indicate that ApCPEB4 and ApCPEB have different RNA binding properties. We tested this idea further by using the CPE site of the 3′UTR of *Aplysia* actin, which is a target mRNA of the ApCPEB [15]. Interestingly, ApCPEB4 did not bind to CPE site in the 3′UTR of *Aplysia* actin, which contains a well-known CPE site (UGUAUUUUUUAUACAAUGUU), whereas ApCPEB showed specific binding to the 3′UTR of actin (Fig. 3c). Instead, ApCPEB4 bound to 1904 U-rich sequence (AAAGAGGAUUUGUGUUUUUCAGGAC), which was designed as a target mRNA for mammalian CPEB3-4 [23] (Fig. 3c). These results suggest that ApCPEB4 is similar to mammalian CPEB3-4 family in its RNA-binding properties. Overall, these results suggest that in its target selectivity ApCPEB4 is functionally closer to the mammalian CPEB3-4 family and is different from ApCPEB.

### ApCPEB4 is critical for the initiation of LTF

**Fig. 4:**
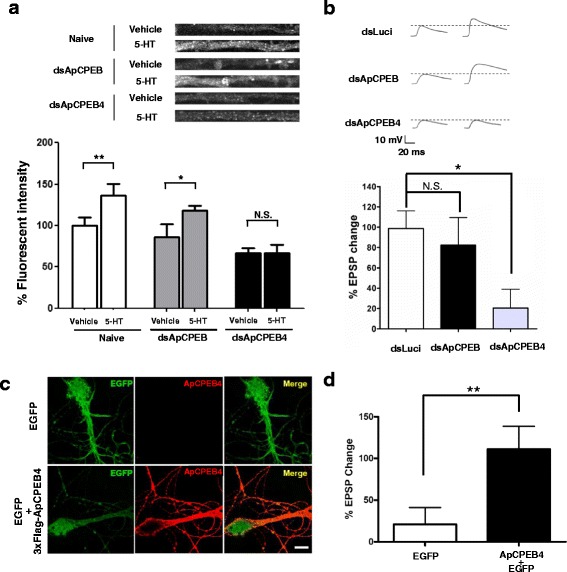
ApCPEB4, but not ApCPEB is critical for the initiation of LTF. **a** The expression and induction of ApCPEB4 following 10 μM 5-HT stimulation was blocked by dsApCPEB4. (*Upper*) Representative images of neurites of cultured sensory neurons immunostained against ApCPEB4. dsApCPEB showed no effect on the ApCPEB4 expression and induction, while dsApCPEB4 significantly blocked ApCPEB4 expression and induction. Scale bar, 40 μm. (Lower) Bar graphs represent the percent fluorescence intensity of ApCPEB4 in the neurites of naïve, dsApCPEB-injected, dsApCPEB4-injected sensory neurons. 5-HT treatment significantly induced the ApCPEB4 expression which was blocked by injection of dsApCPEB4. **, *p* < 0.01; *, *p* < 0.05; N.S., not significant, two-tailed unpaired *t* test. **b** LTF at 24 h was specifically blocked by knock-down of ApCPEB4 (dsApCPEB4). dsApCPEB or dsLuci showed no effect on the 24 h LTF. (*Left*) Representative EPSP traces before and 24 h after the 5 pulses of 5-HT treatment at the sensory-to-motor synapses. (*Right*) Bar graph represents the means ± SEM of the percent change in EPSP amplitude. *, *p* < 0.05 compared with that of dsLuci group, one-way ANOVA; F = 3.83, Tukey’s post-hoc test. N.S. not significant (**c**) Overexpressed 3×Flag-ApCPEB4 in cultured sensory neurons was detected by anti-Flag antibody. As a control, EGFP-expressing sensory neurons were used. Scale bar, 20 μm. **d** The overexpression of ApCPEB4 induced LTF by 1× 5-HT treatment. As a control, EGFP was expressed. Bar graph represents the means ± SEM of the percent change in EPSP amplitude. **, *p* < 0.01, two-tailed unpaired *t* test

Previous reports found that ApCPEB is required for the maintenance of LTF [15]. We thus examined whether ApCPEB4 plays any specific function during LTF in *Aplysia* by knocking down ApCPEB4 transcripts in *Aplysia* sensory neurons. We generated double-stranded (ds) RNAs against N-terminal sequences of ApCPEB (dsApCPEB) and ApCPEB4 (dsApCPEB4). Each ds RNA was injected into cultured sensory neurons, and the protein level of ApCPEB4 in neurites was measured by immunocytochemistry. Baseline expression as well as 5-HT-mediated translation of ApCPEB4 was significantly blocked in neurons injected with dsApCPEB4, but not in the naïve neurons or neurons injected with dsApCPEB (Naïve: no treatment, 100.0 ± 4.9 %, *n* = 26 versus 5-HT treatment, 120.9 ± 5.6 %, *n* = 28; two-tailed unpaired *t* test, ** *p* < 0.01; dsApCPEB: no treatment, 97.1 ± 7.8 %, *n* = 24 versus 5-HT treatment, 119.8 ± 6.3 %, *n* = 21; two-tailed unpaired *t* test,* *p* < 0.05; dsApCPEB4: no treatment, 78.5 ± 5.3 %, *n* = 19 verse 5-HT treatment, 90.4 ± 5.5 %, *n* = 20; two-tailed unpaired *t* test, N.S. (*p* > 0.05)) (Fig. 4a). These data indicate that dsApCPEB4 specifically blocks both endogenous expression and 5-HT-induced expression of ApCPEB4 in *Aplysia* sensory neurons.

We then examined whether ApCPEB4 is required for LTF. Depletion of ApCPEB during 5-HT exposure to 5×5HT blocks the maintenance, beyond 24 h but not the initiation, of the 5-HT-induced LTF [15] during the first 24 h. Interestingly, LTF measured after 24 h was significantly impaired in neurons injected with dsApCPEB4, but not in neurons injected with dsApCPEB or dsLuci (dsLuci, 98.7 ± 17.4 %, *n* = 11; dsApCPEB, 82.3 ± 27.2 %, *n* = 11; dsApCPEB4, 20.5 ± 18.5 % EPSP change, *n* = 12; dsLuci vs. dsApCPEB4, * *p* < 0.05, F = 3.83, one-way ANOVA with Tukey’s post-hoc test) (Fig. 4b), indicating that ApCPEB4 is involved in the initiation of LTF. This result suggests that the regulation of protein synthesis mediated by ApCPEB4 is critical at the initial stage of LTF formation, whereas ApCPEB is critical for the long-term maintenance of LTF.

### Overexpression of ApCPEB4 reduces the threshold of LTF induction

We further examined a specific role of ApCPEB4 in induction of LTF by overexpressing it directly in sensory neurons of sensory-motor cocultures (Fig. 4c). We found that 1× 5-HT (10 μM, 5 min), which normally induces short-term facilitation (STF), induced LTF by overexpression of ApCPEB4, but not EGFP in sensory neurons (EGFP, 21.1 ± 20.4 %, *n* = 11; ApCPEB4 + EGFP, 111.0 ± 27.5 % EPSP change, *n* = 13; two-tailed unpaired t-test, ** *p* < 0.05) (Fig. 4d). These results suggest that the overexpression (artificial induction) of ApCPEB4 reduced the threshold of LTF induction and thus induced LTF with single 5-HT stimulus, further supporting the idea that the translational induction of ApCPEB4 is critical for the formation of LTF in *Aplysia*.

### Phosphorylated ApCPEB4 by PKA is critical for LTF induction

**Fig. 5:**
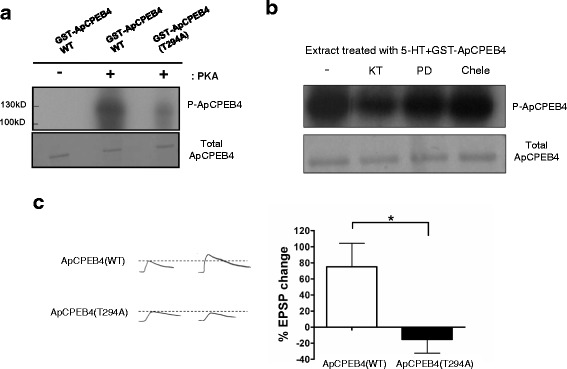
Phosphorylation of ApCPEB4 is required for both the LTF formation. **a** In vitro phosphorylation assay showed that purified ApCPEB4 was directly phosphorylated by PKA on its 294^th^ threonine residue. **b** Phosphorylation of purified ApCPEB4 was examined using *Aplysia* pleural ganglia extracts as an endogenous mixture of kinases. Concurrent treatment of 40 μM KT5720 (KT), a PKA inhibitor, significantly reduced the amount of phosphorylation on ApCPEB4. Neither 20 μM PD98059 (PD), a MEK inhibitor, nor 10 μM chelerythrine (Chele), a PKC inhibitor, affected the phosphorylation of ApCPEB4. **c** Phosphorylation of ApCPEB4 on its 294^th^ threonine residue was required for the LTF formation. (**c**, *left*) Representative traces of EPSP measured at the sensory-to-motor synapses before and 24 h after the 5 pulses of 5-HT. (**c**, *right*) Bar graph represents the mean percentage change ± SEM in EPSP amplitude. Overexpression of ApCPEB4(T294A), non-phosphorylatable mutant of ApCPEB4, significantly blocked LTF. *, *p <* 0.05. two-tailed unpaired *t* test

Previous report showed that ApCPEB is not phosphorylated by PKA [15]. On the other hand, ApCPEB4 possesses one conserved putative PKA phosphorylation site on the 294^th^ threonine residue (Fig. 1a). Thus, we hypothesized that the function of ApCPEB4 might be regulated by PKA-mediated phosphorylation. We first performed an in vitro kinase assay. Purified GST-ApCPEB4 fusion proteins were phosphorylated by the catalytic subunit of PKA in vitro (Fig. 5a). The phosphorylation was reduced in the non-phosphorylatable mutant form of ApCPEB4 (ApCPEB4 T294A), in which 294^th^ threonine was replaced by alanine (Fig. 5a). These results indicate that the 294^th^ threonine of ApCPEB4 is a potential PKA phosphorylation site. In addition, we found that ApCPEB4 was phosphorylated by *Aplysia* neuronal cell lysate in a PKA-dependent manner (Fig. 5b), indicating that ApCPEB4 is a genuine substrate of endogenous PKA in *Aplysia* neurons.

We next asked: Is the phosphorylation of ApCPEB4 by PKA critical for the induction of LTF? If the phosphorylation of ApCPEB4 on the 294th threonine is critical, a mutant form ApCPEB4(T294A) should act as a dominant negative inhibitor. We therefore overexpressed the mutant ApCPEB4 (T294A) in *Aplysia* sensory neurons cocultured with motor neurons and examined the effect of its overexpression on LTF. We found that LTF was completely blocked in the synapse overexpressed with ApCPEB4 (T294A) in sensory neurons, whereas expression of ApCPEB4-WT control had no effect on LTF (ApCPEB4 (WT), 75.0 ± 29.4 %, *n* = 10 versus ApCPEB4 (T294A), −15.4 ± 17.0 % EPSP change, *n* = 5, unpaired t-test, * *p* < 0.05) (Fig. 5c). Taken together, these data indicate that phosphorylation of ApCPEB4 by PKA is required for the induction of LTF in *Aplysia*.

## Discussion

In this study, we cloned a novel protein ApCPEB4, which is related to ApCPEB. Whereas ApCPEB is critical for maintenance, the translational increase of ApCPEB4 was critical for the formation of LTF. Moreover, overexpression of ApCPEB4 reduced the threshold for the LTF. In addition, phosphorylation of ApCPEB4 by PKA was required for the LTF formation. Combined, our results suggest that the two different CPEBs cooperate in different stages during LTF to first initiate and then maintain long-lasting synaptic facilitation.

### ApCPEB4 is essential for the initiation of LTF: different ApCPEBs regulate distinct target mRNAs during LTF

Our data revealed an involvement of ApCPEB4 in the initiation of LTF, and that the overexpression of ApCPEB4 reduces the threshold of LTF induction. This is in contrast to *Aplysia* CPEB, which regulates the maintenance of LTF at 72 h. Thus the two ApCPEBs play distinct roles in 5-HT-induced LTF.

How do these two ApCPEBs regulate LTF formation and maintenance differentially? One plausible explanation is the presence of the prion-like structure in the molecule. The persistence of synaptic plasticity and memory have been found to be mediated by the prion-like CPEB such as ApCPEB in *Aplysia*, orb2 in Drosophila, and CPEB3 in rodent [15, 22, 32]. Synaptic plasticity is mediated by the increase in the aggregation of the prion-like translational regulator ApCPEB or mammalian CPEB3. Therefore, these aggregates serve as functional prions and regulate local protein synthesis necessary for the maintenance of long-term memory. In fact, only antibodies that are specific to the aggregated form block the maintenance of long term facilitation.

Another plausible explanation is that these two ApCPEBs have different RNA binding specificity. We found that ApCPEB but not ApCPEB4 binds to CPE sequence as well as 3′ UTR of actin in CPE-dependent manner (Fig. 3). By contrast, ApCPEB4 bound to a different U-rich sequence, the 1904 sequence, which is a synthetic binding sequence for mammalian CPEB3-4, but not a canonical CPE (Fig. 3) [23]. In fact, mammalian CPEB1 and mammalian CPEB2-4 also have different target mRNAs to regulate translation for different stages of synaptic plasticity via CPE site-dependent and-independent manners, respectively [23]. In contrast to our results, it has been reported that mammalian CPEB4 seems to be dispensable for hippocampus-dependent plasticity and learning and memory [33]. However, unlike to *Aplysia* and Drosophila, which have two types of CPEB, mammalian has four CPEB family including CPEB1-4, which might compensate other CPEBs.

These observations, suggest that activated ApCPEB and ApCPEB4 may regulate protein synthesis of two distinct groups of mRNAs, one group of mRNAs containing CPE sites for the maintenance of LTF and another group mRNAs containing CPE-independent sites for the initiation of LTF. It would be interesting to further discriminate target mRNAs used for distinct phases of LTF that are translated by ApCPEB and ApCPEB4, respectively.

### PKA-dependent activation of ApCPEB4

In *Xenopus* oocytes, CPEB1 is phosphorylated by the kinase Aurora A (Eg2) at a canonical LD (S/T)R site [34, 35], and the phosphorylation of CPEB1 binds to cleavage and polyadenylation specificity factor (CPSF) to induce release of PARN from the ribonucleoprotein (RNP) complex, thereby enabling Germ-line-development factor 2 (Gld2) to elongate poly(A) tailing by default [35]. On the other hand, ApCPEB has been found not to be phosphorylated but to be increased in the amount of protein expression to enhance the affinity to CPSF [15]. Interestingly, ApCPEB4 is regulated differentially from ApCPEB. ApCPEB4 is directly phosphorylated by PKA on its canonical LD(S/T)R site.

In *Aplysia*, PKA is critical for both synapse-specific and cell-wide facilitation induced by 5-HT signaling. PKA phosphorylates many components required for LTF formation in *Aplysia* such as cAMP response element-binding protein (CREB), synapsin, *Aplysia* Activating Factor (ApAF), and Cell Adhesion Molecule-Associated Protein (CAMAP) [36–40]. Although we do not provide direct evidence, our data provide further insight into the mechanism of how the long-lasting forms of synaptic plasticity can be initiated via PKA-mediated phosphorylation and local translation of ApCPEB4. ApCPEB4 might connect PKA signaling to the local protein synthesis, which is required for the induction of more sustained synaptic activation, by means of the enhanced expression of target mRNAs of ApCPEB4 to support 5-HT-induced LTF.

### Possible roles of ApCPEB4 in synapse-specific LTF

As shown in Fig. 2, ApCPEB4 protein can be localized in neurites. In addition, we previously reported that ApCPEB4-EGFP could form RNA granules within the neurites in *Aplysia* sensory neurons [41]. Combined, ApCPEB4 can be localized in neurites and involved in local protein synthesis.

During synapse-specific LTF, local protein synthesis is required for two distinct phases of LTF: initiation and maintenance [8, 31]. For the maintenance of synapse-specific LTF, a rapamycin-sensitive local protein synthesis is required [8, 31]. One essential molecule which is locally synthesized in a rapamycin-sensitive manner is ApCPEB. ApCPEB regulates local translation of many specific mRNAs containing CPE sites including actin mRNA to sustain the synaptic facilitation for periods up to 72 h by supporting persistent structural and functional changes of the synapses [42]. However, for the initiation of LTF, a second, rapamycin-insensitive, emetine-sensitive component of local protein synthesis is required in synapse-specific LTF [31]. Our data illustrate that local induction of ApCPEB4 by 5-HT treatment is rapamycin-insensitive and emetine-sensitive. In addition, we also found that one pulse of 5-HT produced LTF in ApCPEB4-overexpressing sensory neurons. It is therefore possible that overexpression of ApCPEB4 combined with one pulse of 5-HT may be sufficient to produce the retrograde signal required for LTF induction. Overall, ApCPEB4 may be a key regulator required for generating the retrograde signal in initial local protein synthesis during synapse-specific LTF. Although it is still possible that ApCPEB4 may be involved in the rapamycin-insensitive, emetine-sensitive intermediate-term facilitation (ITF) [43, 44], it would be interesting to further dissect this possibility in a synapse-specific form of LTF.

In this study, we investigated the molecular and cellular function of a novel CPEB isoform in *Aplysia*, ApCPEB4. ApCPEB4 was translated and increased by stimuli inducing LTF and is required for the formation of LTF. Overexpression of ApCPEB4 reduced the threshold for LTF induction, and phosphorylation of ApCPEB4 by PKA was critical for the induction of LTF. ApCPEB4 and ApCPEB have distinct RNA binding selectivity: ApCPEB4 did not bind to the CPE sequence in actin mRNAs to which ApCPEB binds, whereas ApCPEB4 bound to non-CPE U-rich RNA sequence that was a target of mammalian CPEB2-4. Taken together, these results indicate ApCPEB4 plays a key role in the initiation of LTF in *Aplysia*, in parallel with the key role ApCPEB has in the maintenance of LTF.

## Additional file

Additional file 1:
**Figure S1**. A representative Western blot (left) and quantification (right) of ApCPEB4 in *Aplysia* pleural ganglia extracts prepared from animals exposed to 5-HT in vivo for 2 h. Total extracts were prepared at indicated times and 20 μg of proteins were blotted with anti-ApCPEB4 antibodies (left, top panel). The same extracts were also stained with Coomassie blue as loading controls (left, bottom panel). (PDF 49 kb)

## Abbreviation

3′UTR: 3′ untranslated region
5-HT: 5-hydroxytryptamine
ApAF: *Aplysia* activating factor
ApC/EBP: *Aplysia* CCAAT-enhancer-binding proteins
CAMAP: Cell adhesion molecule-associated protein
CNS: Central nervous system
CPE: Cytoplasmic polyadenylation element
CPEB: Cytoplasmic polyadenylation element binding protein
CPSF: Polyadenylation specificity factor
CREB: cAMP response element-binding protein
EST: Expression sequence tag
Gld2: Germ-line-development factor 2
ITF: Intermediate-term facilitation
LTF: Long-term facilitation
LTP: Long-term potentiation
PABP: The poly(A) binding protein
PARN: Element contain poly A ribonuclease
PKA: Protein kinase A
RNP: Ribonucleoprotein
RRM: RNA recognition motif
RT-PCR: Reverse transcription-polymerase chain reaction
STF: Short-term facilitation
