# Supplementary figures and images for: ApCPEB4, a non-prion domain containing homolog of ApCPEB, is involved in the initiation of long-term facilitation
Source: Mol Brain. 2016 Oct 22;9:91. doi: 10.1186/s13041-016-0271-x (PMC5075418; doi:10.1186/s13041-016-0271-x)

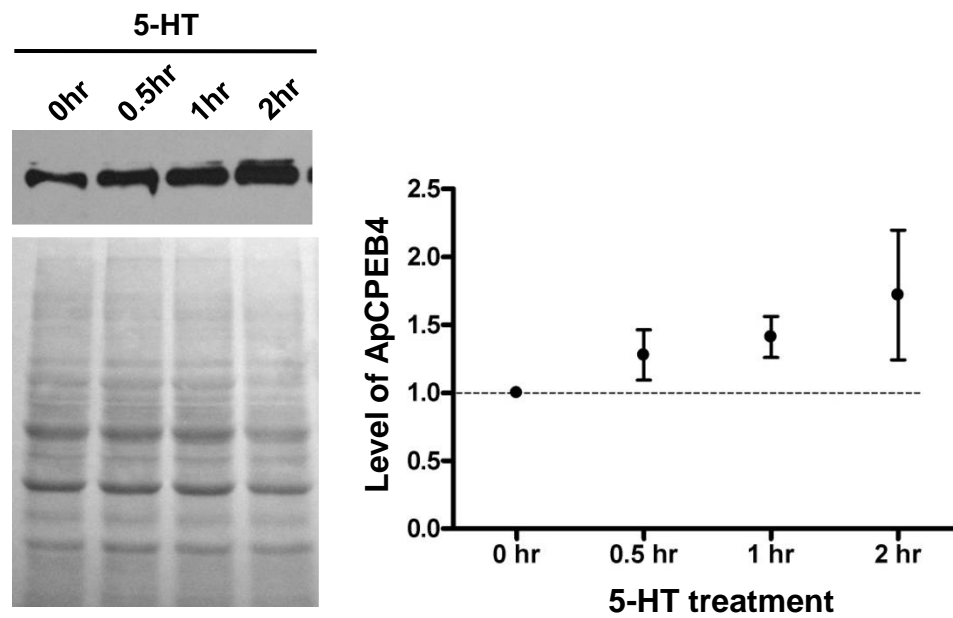

Supplementary Fig. 1

Supplement: Additional file 1: — Figure S1. A representative Western blot (left) and quantification (right) of ApCPEB4 in Aplysia pleural ganglia extracts prepared from animals exposed to 5-HT in vivo for 2 h. Total extracts were prepared at indicated times and 20 μg of proteins were blotted with anti-ApCPEB4 antibodies (left, top panel). The same extracts were also stained with Coomassie blue as loading controls (left, bottom panel). (PDF 49 kb) [file 13041_2016_271_MOESM1_ESM.pdf]
